# Single-Crystalline Nanowires of Molecular Ferroelectric Semiconductors for Optoelectronic Memory

**DOI:** 10.3390/nano14231920

**Published:** 2024-11-28

**Authors:** Xinxia Qiu, Mingsheng Xu, Chunxiao Cong, Zhi-Jun Qiu, Laigui Hu, Ran Liu

**Affiliations:** School of Information Science and Technology, Fudan University, Shanghai 200433, China; 21210720051@m.fudan.edu.cn (X.Q.); 18110720080@fudan.edu.cn (M.X.); cxcong@fudan.edu.cn (C.C.); zjqiu@fudan.edu.cn (Z.-J.Q.); rliu@fudan.edu.cn (R.L.)

**Keywords:** molecular ferroelectric semiconductor, single-crystalline nanowires, self-powered optoelectronic devices, ferroelectric memory

## Abstract

Though much progress has been achieved in the discovery of new molecular ferroelectrics in recent years, practical applications and related physics are still rarely explored due to the difficulty in high-quality film production and patterning issues. Single-crystalline films and patterns are in high demand for high device performance. Through a template-assisted space-confined strategy, herein, ordered single-crystalline nanowire patterns and optoelectronic devices of a semiconducting molecular ferroelectric (SMF), hexane-1,6-diammonium pentaiodobismuth (HDA-BiI_5_), were successfully demonstrated. The coupling of semiconducting and ferroelectric polarization of the SMF devices enables a broadband self-powered photodetection from ultraviolet to visible light, as well as polarization-tunable photoresponsivity. These may open an avenue for high-performance SMF optoelectronic memory devices with low cost and flexibility.

## 1. Introduction

With the advantages of low cost, flexibility, ease of processing, and large-area production, organic ferroelectrics show a high promise for various functional devices, such as memory and piezoelectric devices [1,2,3,4]. Unfortunately, only ferroelectric polymer polyvinylidene fluoride (PVDF) and its copolymers were widely and deeply investigated [5,6,7]. Poor ferroelectricity, including large coercive electric fields and crystalline film morphology, significantly hinders their practical applications [3,8,9]. During the past two decades, the discovery of various molecular ferroelectrics (MFs) with excellent properties has provided an alternative source for flexible and low-cost ferroelectric devices [2,10,11,12,13]. However, their feasibility for the applications in memory devices, as well as fundamental physics, are still sparsely explored and necessitate further investigation. High-quality films or even single-crystalline films and patterns are usually excessively demanded, which can naturally yield high device performance [14,15,16,17].

Among the MFs, semiconducting MFs (SMFs) are specifically fascinating owing to their high potential for self-powered flexible optoelectronic and memory devices. The coupling between ferroelectric polarization and semiconducting properties can endow SMFs with tunable photosensitivity by polarization [18,19,20,21]. A ferroelectric-induced built-in electric field can be employed for the separation of photogenerated electron-hole pairs in various optoelectronic devices [18,22,23]. Specifically, it was considered that the conversion efficiency of ferroelectric photovoltaics could break from the Shockley-Queisser limit [24,25,26,27]. For instance, hexane-1,6-diammonium pentaiodobismuth (HDA-BiI_5_), an n-type SMF with remnant polarization of 6.2 μC/cm^2^ and coercive field of about 9.3 kV/cm at 358 K, was recently applied in perovskite solar cells [26,28], flexible/wearable electronics, and ferroelectric photovoltaic devices [13,29,30]. Though it shows a high potential with broadband absorption from ultraviolet to visible wavelengths, the employed large bulk crystals or polycrystalline spin-coated films significantly limit further development for device applications. To make full use of the ferroelectric properties of the SMFs, single-crystalline devices are always preferred, which can also yield better charge transport [29,31,32]. However, the achievement of single-crystalline films/patterns and devices is still a challenge, as well as for the other MFs [33,34,35].

In this study, ordered single-crystalline nanowires (or nanorods) and devices of HDA-BiI_5_ were successfully developed using a self-assembly method, i.e., a template-assisted space-confined strategy. Using a Si template with micropillars, rod-like single crystals or wires were in situ grown with their widths ranging from hundreds of nanometers to micrometers. It is notable that the polar c-axis of the SMF is parallel to the long-axis direction of the nanowires, implying a potential for the full use of remnant ferroelectric polarization. With these nanowires, lateral capacitive memories were fabricated, which can exhibit reconfigurable self-powered photoresponse controlled by ferroelectric polarization. Self-powered polarization-tunable photoresponses were then demonstrated at 0 V, which implies a high potential for low-cost flexible SMF-based photomemories.

## 2. Materials and Methods

### 2.1. Materials

Octadecyltrichlorosilane (OTS) was purchased from Sigma-Aldrich Corporation without further purification. All the other reagents were of analytical grade and commercially obtained. HDA-BiI_5_ was synthesized based on the reported literature [13]. See experimental details in Figure S1.

### 2.2. Fabrication and Modification of the Silicon Template

The Si template with a micropillar structure was fabricated as follows: (1) Double-layer photoresist (Shipley Microposit Lor 5B and S1800 series) films with ca. 1 μm thickness were spin-coated on a cleaned four-inch silicon wafer. (2) A direct laser writing apparatus (Heidelberg DWL200) was used to perform strip pattern lithography on the silicon wafer. (3) Deep reactive ion etching (DRIE, Alcatel 601E) for the template was then carried out using fluorine-based reagents for 8 min. (4) After removing the photoresist, the micropillar structure was obtained with a width of 1 μm, a gap of 10 μm, and a height of 20 μm (see Figure S2a). (5) The silicon template was cleaned with acetone, isopropyl alcohol, and deionized water and then selectively treated with OTS to obtain asymmetric wettability micropillars, resulting in hydrophobic sidewalls and hydrophilic tops of the micropillar template. Specifically, the silicon template with photoresist-protected tops of the micropillars was exposed to OTS vapor at 90 °C for 4 h. See Figure S3 for more details of the asymmetric wettability modification of the template with OTS.

### 2.3. Growth of HDA-BiI_5_ Nanowires

To form single-crystal molecular ferroelectric films, the silicon template with micropillar structures was employed to guide the formation of the HDA-BiI_5_ nanowires. A precursor solution was prepared by dissolving HDA-BiI_5_ in a coordination solvent dimethyl sulfoxide (DMSO), which is conducive to the slow crystallization of HDA-BiI_5_ for a high crystallinity and preferred orientation film [36,37]. The substrate was washed with acetone, ethanol, and deionized water in turn and finally dried with a nitrogen stream before use. The target substrate was then placed onto a Si template to construct a sandwich structure, forming an asymmetrically wetted mold. The HDA-BiI_5_ solution (10 mg/mL, 10 μL) pre-injected between the template and the substrate gradually evaporated and crystallized during heating on a hot plate at 60 °C in a nitrogen glove box for 48 h, leaving the nanowires on the substrate.

### 2.4. Characterizations of the HDA-BiI_5_ Nanowires

The prepared HDA-BiI_5_ nanowires were then characterized by optical microscopy (OM, VHX-600E), cross-polarized optical microscopy (CPOM, Olympus BX53), scanning electron microscope (SEM, Zeiss Sigma HD), atomic force microscope (AFM, Park System NX10), X-ray diffractometry (XRD, Bruker AXS D8), transmission electron microscopy (TEM, FEI Tecnai G2 F20), UV-Vis absorption spectrometer (Lambda 750S), and fast Raman imaging spectrometer (SHG, WITec, alpha300).

### 2.5. Device Fabrication and Measurements

After obtaining the single-crystalline HDA-BiI_5_ nanowires, a pair of Au/Cr electrodes with a gap of 100 μm were deposited using vacuum deposition through a shadow mask. The electrodes are perpendicular to the nanowires, forming a top contact structure. Photocurrent and electric characterizations were conducted by using a B1500A semiconductor analyzer with the light source from a microscopic confocal laser Raman spectrometer (Renishaw inVia).

## 3. Results and Discussion

**Figure 1:**
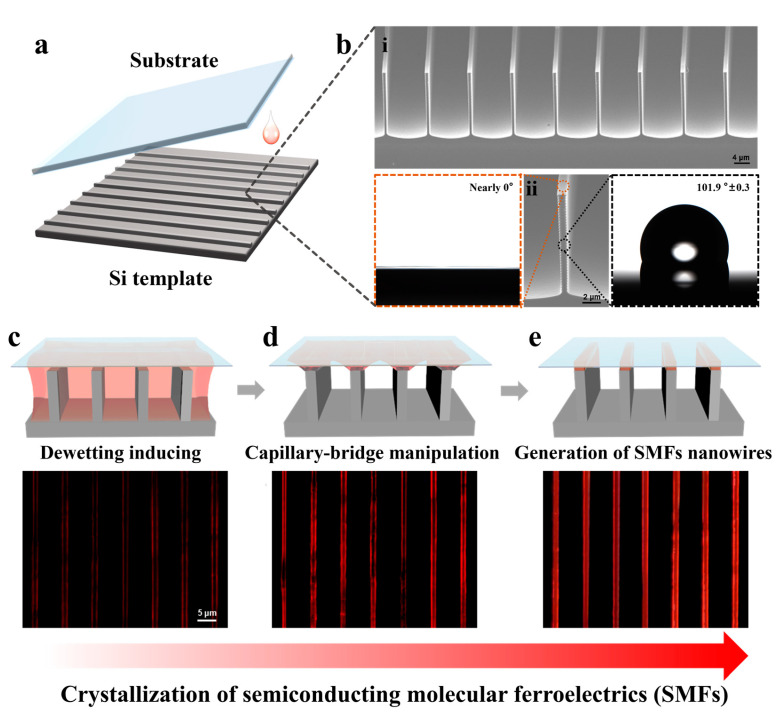
Growth of HDA-Bil_5_ crystalline nanowires. (**a**) Fabrication process for semiconducting molecular ferroelectrics HDA-BiI_5_ crystalline nanowires: a template-assisted space-confined method. (**b**) The selectively surface-modified silicon template: (**i**) SEM image of the silicon template and (**ii**) enlarged SEM image of the micropillar structure. Left inset: the solution contact angle of the lyophilic tops of the micropillars. Right inset: the solution contact angle of the hydrophobic sidewalls. (**c**–**e**) Schematic diagram and in situ CPOM images of the crystallization of the SMFs.

As shown in Figure 1a, HDA-BiI_5_ nanowires were successfully fabricated using the template-assisted space-confined method. Figure 1(bi) is a detailed SEM image of the silicon template with micropillars (width 1 μm, spacing 10 μm). Figure 1(bii) is an enlarged SEM image of a single micropillar structure. To confirm the asymmetric wettability structure, static contact angle tests were performed. After asymmetric-wetting modification of the silicon template, the solution contact angle (CA) of the lyophilic tops of the micropillars is estimated to be 0° (see the left inset), while the CA of the hydrophobic sidewalls is 101.9° ± 0.3°, as shown in the right inset. In such an asymmetric wettability structure, the pre-injected liquid will be repelled from gaps between the micropillars towards the top of the micropillars and evaporated, resulting in directional capillary flow to compensate for the loss of solution [8,33,38]. This can be attributed to the construction of discrete capillary bridges, leading to the eventual formation of nanowires under dewetting dynamics [39].

The growth process of molecular ferroelectric crystalline HDA-BiI_5_ nanowires can be in situ observed using CPOM. Initially, ferroelectric molecules are found to preferentially nucleate on both sides of the capillary bridge (Figure 1c), due to high local concentrations and rapid evaporation at the contact lines of the capillary bridge [38]. The concentration gradient then drives the directional transport of the solute to the interior of the capillary bridge, leading to further coarsening of the crystals (Figure 1d). A pair of nanowires with an increasing width (hundreds of nanometers) for each capillary bridge can be observed. Finally, such a pair of crystalline nanowires merges into a single-oriented nanowire after the complete evaporation of solvents (Figure 1e). Therefore, the template-assisted space-confined method has proved to be an effective strategy for guiding the HDA-BiI_5_ crystal growth.

**Figure 2:**
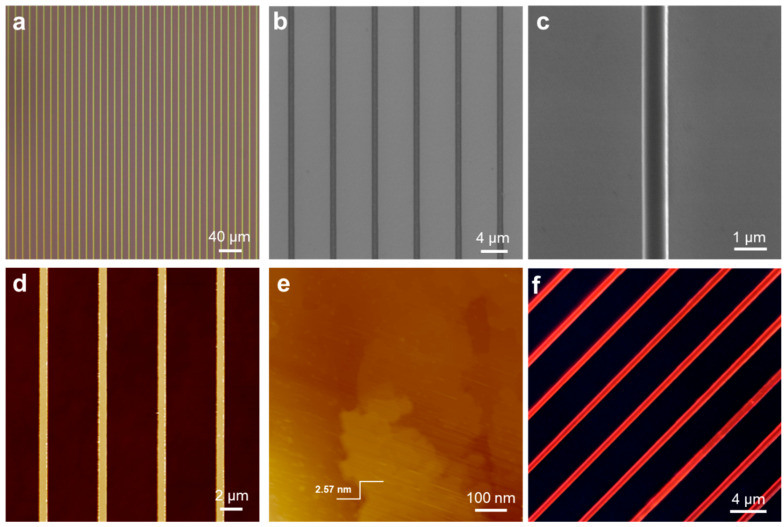
The morphology of molecular ferroelectric semiconductor nanowires. (**a**) Optical microscope images of the large-area HDA-BiI_5_ nanowires. (**b**) SEM image of the nanowires. (**c**) Enlarged SEM image. (**d**) AFM image of the nanowires. (**e**) AFM morphology image of the HDA-BiI_5_ nanowires. (**f**) CPOM image of the patterned HDA-BiI_5_ nanowires.

Figure 2a shows an optical microscope image of a large-area HDA-BiI_5_ nanowire array, displaying a well-organized and highly ordered microstructure. To eliminate optical interference, SEM and AFM were used to further characterize the nanowires. The SEM image shows that HDA-BiI_5_ nanowires were aligned directionally with uniform size and precise growth localization (Figure 2b). The enlarged SEM image further confirmed uniform nanowires with smooth surfaces, sharp edges, dense interiors, and invisible grain boundaries (Figure 2c). The AFM image further confirms that the HDA-BiI_5_ nanowires exhibit microscopic uniformity, with a width of approximately 800 nm (Figure 2d). Moreover, the crystalline wires exhibit tunable dimensions, ranging from nanoscale to microscale widths, which are determined by the silicon templates with micropillars of varying widths, as detailed in Figure S2. Figure 2e shows the AFM topography of a nanowire surface on which a layered structure with a roughness of only 2.57 nm was observed. The AFM height curve indicates that the nanowires exhibit a uniform height of approximately 350 nm (Figure S4). The CPOM image of the nanowires was also collected (Figure 2f), which exhibits uniform luminous intensity, indicating the high possibility of single-crystalline features.

**Figure 3:**
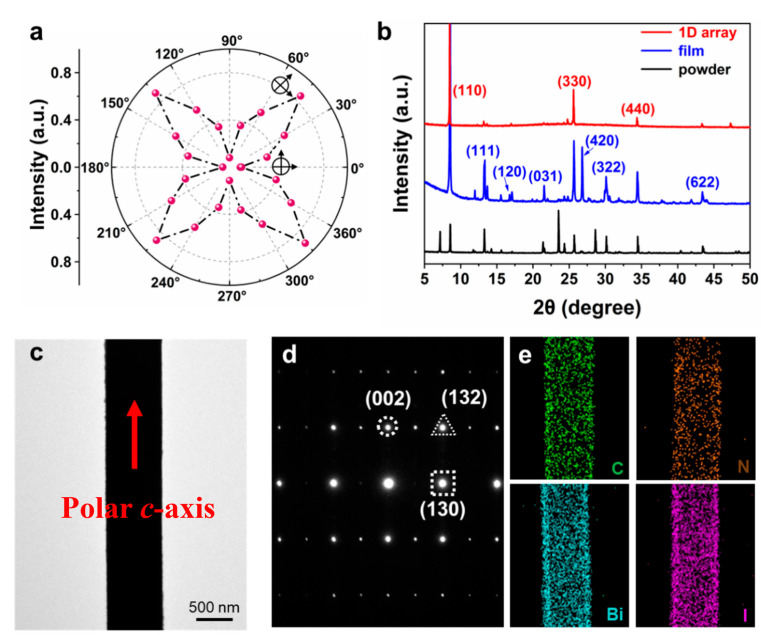
Crystallographic structure and polarization orientation of HDA-BiI_5_ single crystal nanowires. (**a**) Polarizing angle dependence of normalized reflection intensity from the HDA-BiI_5_ nanowires. (**b**) Out-of-plane XRD patterns of the 1D HDA-BiI_5_ single crystal nanowire arrays, spin-coated film, and powders on glass substrates. (**c**) TEM topography of a single HDA-BiI_5_ nanowire. (**d**) The corresponding SAED pattern and (**e**) EDS image.

Further investigation of the crystallographic orientation and crystallinity of the HDA-BiI_5_ nanowires was then conducted. By rotating the sample every 15 degrees, polarizing angle dependence of normalized reflection intensity under CPOM from the nanowires was obtained, as shown in Figure 3a. A fourfold symmetric brightness pattern due to a high anisotropy of the crystalline wires was observed. Then, different diffraction patterns of HDA-BiI_5_ were characterized by using out-of-plane XRD, as shown in Figure 3b. The XRD pattern of HDA-BiI_5_ powder (black line) exhibits multiple diffraction peaks, representing a disordered arrangement of multiple polymorphs, while the spin-coated film of HDA-BiI_5_ (blue line) also shows characteristics of a polycrystalline thin film. In contrast to the powder and spin-coated film, the XRD spectrum of HDA-BiI_5_ nanowires demonstrates a series of periodic sharp diffraction peaks, corresponding to the (*l*00) lattice plane diffraction of the 2D layered HDA-BiI_5_ (red line), confirming their single-crystalline orientation.

Moreover, TEM and selected area electron diffraction (SAED) were used to characterize the morphology and crystallography of the HDA-BiI_5_ nanowires. Figure 3c is a TEM topography image of a single HDA-BiI_5_ nanowire with sharp edges. No grain boundaries or cracks were observed. The corresponding SAED pattern (Figure 3d) exhibits a set of sharp diffraction spots, which can be indexed to the (002), (130), and (132) lattice planes, indicating that the crystals growing along the direction (002) have monocrystalline properties. The polar axis (*c*-axis) of the HDA-BiI_5_ single-crystalline microwires was revealed to be aligned along the long axes of the nanowires (Figure 3c), illustrating the capability of in-plane ferroelectric polarization. The elemental composition of the single HDA-BiI_5_ nanowire was attained by corresponding energy-dispersive X-ray spectroscopy (EDS) element mappings. As shown in Figure 3e, the distribution of each element (C, N, Bi, and I) is uniform and conforms to the HDA-BiI_5_ chemical formula.

**Figure 4:**
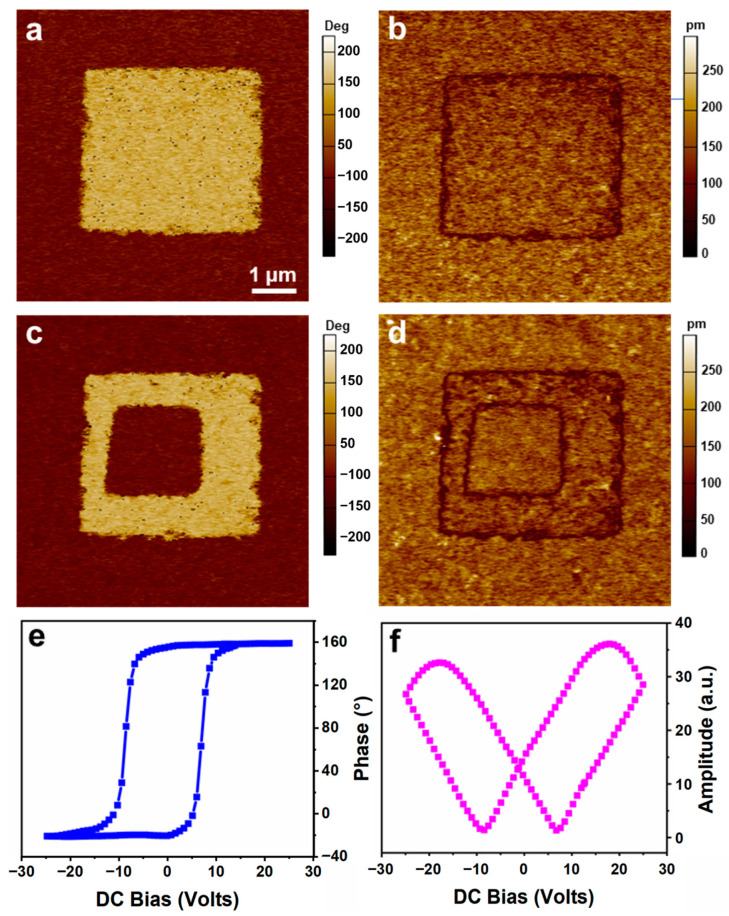
Ferroelectricity of the HDA-BiI_5_ single-crystalline nanowires. Lateral PFM phase (**a**,**c**) and amplitude (**b**,**d**) images of HDA-BiI_5_ single crystals. (**e**) Phase–voltage hysteresis loop (blue line). (**f**) Amplitude-voltage butterfly loop (red line).

To verify the ferroelectricity, piezoelectric response force microscopy (PFM) was then adopted for characterization. A tip bias voltage of -10 V was employed for the polarization of a randomly selected single-crystalline HDA-BiI_5_ (4 μm × 4 μm), while a positive voltage of +10 V was applied for the remaining regions. As expected, the lateral PFM (LPFM) phase image (Figure 4a) shows two different regions with a 180° phase contrast, and the amplitude image (Figure 4b) presents the clear domain walls of the two domains with different orientations. Similarly, opposite domains were produced in a smaller square area in the middle, as shown in Figure 4c,d, resulting in another well-defined square pattern. In addition, a perfect phase–voltage hysteresis loop (Figure 4e) and amplitude-voltage butterfly loop (Figure 4f) can be obtained by applying a point voltage locally through a PFM probe. The local coercive voltage (*V*_c_) at a point in the film can be estimated to be ~9 V. Therefore, the domains of HDA-BiI_5_ single crystals can be patterned and show 180-degree polarization reversal.

As reported in Ref. [13], the ferroelectricity originates from the breakdown of the inversion symmetry of its crystal structure. The molecular ferroelectric HDA-BiI_5_ crystal structure consists of one-dimensional zigzag chains of corner-sharing BiI_6_ octahedra and hexane-1,6-diammonium cations, exhibiting a polar *Pna*2_1_ orthorhombic structure (ferroelectric phase). It will transform into the centrosymmetric space group *Pnam* structure when the temperature exceeds its Curie temperature (393K). It is worth mentioning that the ammonium heads of the cation are arranged along the *c*-axis, which is the polar axis of the crystal [13]. Since the direction of the growth axis of the nanowire is parallel to the polarization *c*-axis, it can ensure the potential for the full use of remnant polarization along the nanowires.

**Figure 5:**
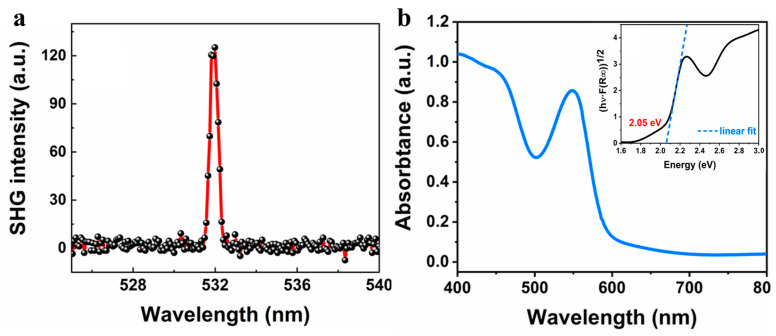
Optical properties of HDA-BiI_5_ single crystal nanowires. (**a**) SHG signal from the HDA-BiI_5_ single crystal. (**b**) Absorption spectrum of the HDA-BiI_5_ nanowires and its corresponding Tauc plot (inset).

To further understand the ferroelectricity of the SMF, the optical second harmonic generation (SHG) effect was investigated, which can verify the breakdown of inversion symmetry in crystals (a prerequisite for ferroelectricity) [11,40]. A laser wavelength of 1064 nm was employed to detect the SHG signals, and a distinct signal peak at 532 nm was observed (Figure 5a). This confirms that the HDA-BiI_5_ crystals demonstrate inversion symmetry breaking with the polar *Pna*2_1_ orthomorphic structure, consistent with the structural characteristics of ferroelectrics. In addition to their excellent ferroelectric properties, the optical properties of the HDA-BiI_5_ nanowires were also investigated. Ultraviolet-visible (UV-Vis) absorption spectra of the HDA-BiI_5_ crystal nanowires on a transparent glass substrate were characterized as shown in Figure 5b, illustrating strong absorption starting at about 650 nm, which is consistent with the reported value in the literature [30]. The corresponding Tauc plot was demonstrated as the inset. A bandgap of 2.05 eV can be extracted from the absorption edge, implying a broad-spectrum absorption of HDA-BiI_5_ from ultraviolet to visible regions. The narrow bandgap suggests that single-crystalline nanowires could be utilized for photoresponsive devices.

**Figure 6:**
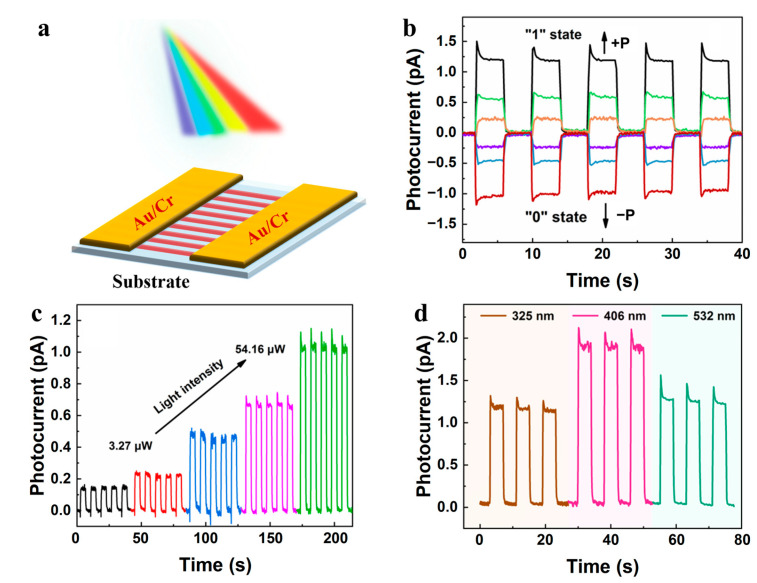
Capacitive optoelectronic memory devices based on the HDA-BiI_5_ single crystal nanowires. (**a**) Schematic diagram of the HDA-BiI_5_ nanowire photoelectric device. (**b**) The photocurrent under different light intensities at 325 nm and 0 V after poling using different voltage polarities. (**c**) The tunability of the device to store information under different powers at 325 nm wavelength. (**d**) Photocurrent with different wavelengths (325 nm, 406 nm, and 532 nm) of light at 0 V.

Capacitive optoelectronic memory devices of the single-crystalline HDA-BiI_5_ nanowires were then constructed, as shown in the schematic illustration shown in Figure 6a. Their electrical performance under illumination was investigated before and after poling. To polarize the device, a voltage of 30 V was applied. As expected, no photocurrent was observed for the device before poling. In contrast, a distinct photocurrent from the SMF device after poling was observed at 0 V upon illumination. Considering the symmetric electrodes, a built-in electric field generated after poling should be responsible for the exciton dissociation and charge transport even without applying a bias voltage. Negative poling was also applied to the device. It is noted that the polarities of the photocurrent can be reversed by changing the poling direction, implying optoelectronic memory functions with the memory states of 0 and 1, respectively (see Figure 6b). Therefore, the stored information in the HDA-BiI_5_ single-crystalline device can be read by detecting the polarities of photocurrent. Figure 6c specifically illustrates that the magnitude of photocurrent varies with the change in light intensity. Higher intensity can induce a large photoresponse. The photoresponses of the device under a state of ‘1’ at 0 V also show independence with light illumination wavelengths (see Figure 6d). Benefiting from the small band gap (~2.05 eV) of the SMF, distinct photocurrent can be induced by visible light, in contrast to other insulating and transparent ferroelectrics.

The distinct photoresponse after poling clearly indicates the semiconducting property of HDA-BiI_5_, which can be tunable by ferroelectric polarization switching. The polarization-induced electric field inside the device is responsible for the charge separation and photocurrent generation. In addition, the polarities of photocurrent can be changed by polarization reversal through poling, implying the coupling between ferroelectric polarization and semiconducting properties. It enables the potential of the SMF single-crystalline device for broadband optoelectronic memory applications, even for self-driven non-volatile memories.

In addition, the device’s performance over multiple switching cycles exposed to light for long periods of time was also investigated, which is critical to evaluating its potential applications. As shown in Figure S5, there was no significant decrease in photocurrent after reading the device multiple times for 2000 s, which can be attributed to the consistent orientation, single-crystallinity, and high uniformity of the HDA-BiI_5_ nanowires. Therefore, the simple capacitor structure for the SMF nanowire-based devices can achieve a stable optoelectronic memory function.

## 4. Conclusions

In summary, we have obtained ordered single-crystalline nanowire arrays of the molecular ferroelectric semiconductor (HDA-BiI_5_) through a template-assisted space-confined method. The single crystallinity of the HDA-BiI_5_ nanowires was confirmed through CPOM, XRD, TEM, and SAED measurements. Ferroelectricity was further verified by PFM measurements. It is noted that the polarization direction of the single crystalline nanowires is parallel to the long axis, enabling in-plane ferroelectric memory devices. Due to the narrow bandgap and wide absorption spectrum of HDA-BiI_5_, single-crystalline nanowire-based optoelectronic memory devices were then successfully demonstrated with tunable photoresponsivity. With the coupling of ferroelectric polarization and semiconducting properties, the achievements of single-crystalline SMF nanowires may lay the foundation for self-powered photomemory devices.

## Data Availability

The research data of this work are available upon request to authors.

## Supplementary Materials

The following supporting information can be downloaded at: https://www.mdpi.com/article/10.3390/nano14231920/s1, Figure S1: Synthesized crystals of HDA-BiI_5_; Figure S2: SEM images of micropillars on silicon templates with a width of 1 μm and a gap of 10 μm (a) and the corresponding prepared HDA-Bil_5_ single crystalline nanowires (c). Micropillars with a width of 4 μm and a gap of 10 μm (b) and the corresponding prepared HDA-Bil_5_ single crystalline nanowires (d); Figure S3: Schematic diagram for the asymmetric wettability modification of silicon templates with OTS; Figure S4: (a) AFM image of the HDA-BiI_5_ single-crystalline nanowire arrays and (b) corresponding height curves; Figure S5: Endurance of capacitive optoelectronic memory devices based on the HDA-BiI_5_ single crystal nanowires.
