# Supplementary material for: Single-Crystalline Nanowires of Molecular Ferroelectric Semiconductors for Optoelectronic Memory
Source: Nanomaterials (Basel). 2024 Nov 28;14(23):1920. doi: 10.3390/nano14231920 (PMC11643503; doi:10.3390/nano14231920)
Supplement: Supplementary file 1 [file nanomaterials-14-01920-s001.zip › nanomaterials-3302401-supplementary.pdf]

nanomaterials

Supplementary Materials

Single-crystalline nanowires of molecular ferroelectric semiconductors for optoelectronic memory

Xinxia Qiu[&], Mingsheng Xu[&], Chunxiao Cong, Zhi-Jun Qiu, Laigui Hu*, Ran Liu

School of Information Science and Technology, Fudan University, Shanghai 200433, China; 21210720051@m.fudan.edu.cn (X.Q.); 18110720080@fudan.edu.cn (M.X); cxcong@fudan.edu.cn (C.C.); zjqiu@fudan.edu.cn (Z.-J.Q.); rliu@fudan.edu.cn (R.L.)
[&] These authors contributed equally to this work.
* Correspondence: laiguihu@fudan.edu.cn

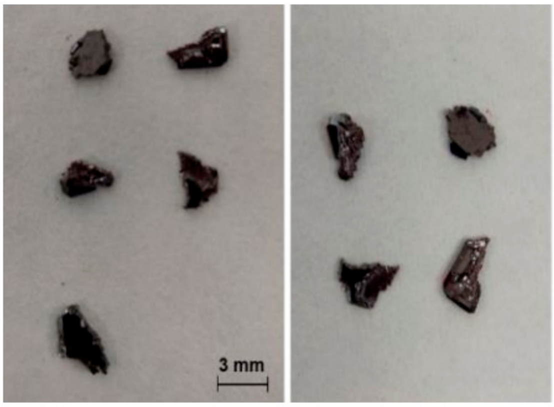

Figure S1. Synthesized crystals of HDA-BiI_5. The HDA-BiI_5 crystals were prepared by cooling a hot saturated solution. The specific synthesis steps were as follows: BiI_3 (5.0 mmol, 2.95 g) powders and hydroiodic acid aqueous solution (50 ml, 45 wt%) were respectively added into a round-bottom flask, and stirred until the iodide medium was completely dissolved. The flask was heated in a water bath to 363 K, and then $H_2N(CH_2)_6NH_2$ (5.0 mmol, 0.58 g) was added. Condensation reflux reaction was carried out and the temperature was slowly cooled to room temperature. Finally, the solution was filtered to obtain $[NH_3(CH_2)_6NH_3]BiI_5$ (HDA-BiI_5) bulk crystals with dark red color. The whole reaction process was carried out in a nitrogen atmosphere.

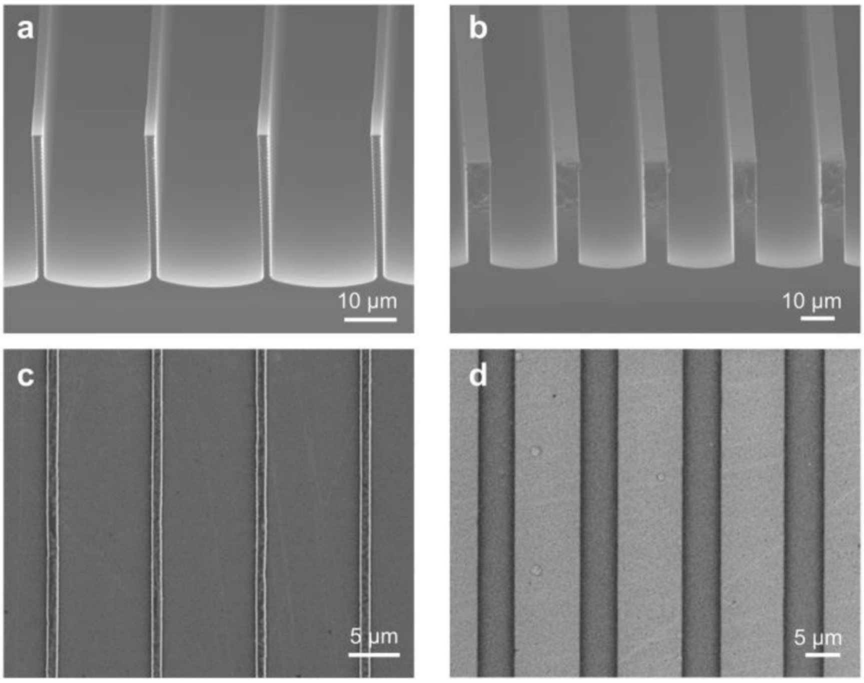

Figure S2. SEM images of micropillars on silicon templates with a width of 1 μm and a gap of 10 μm (a) and the corresponding prepared HDA-BiI_5 single crystalline nanowires (c). Micropillars with a width of 4 μm and a gap of 10 μm (b) and the corresponding prepared HDA-BiI_5 single crystalline nanowires (d).

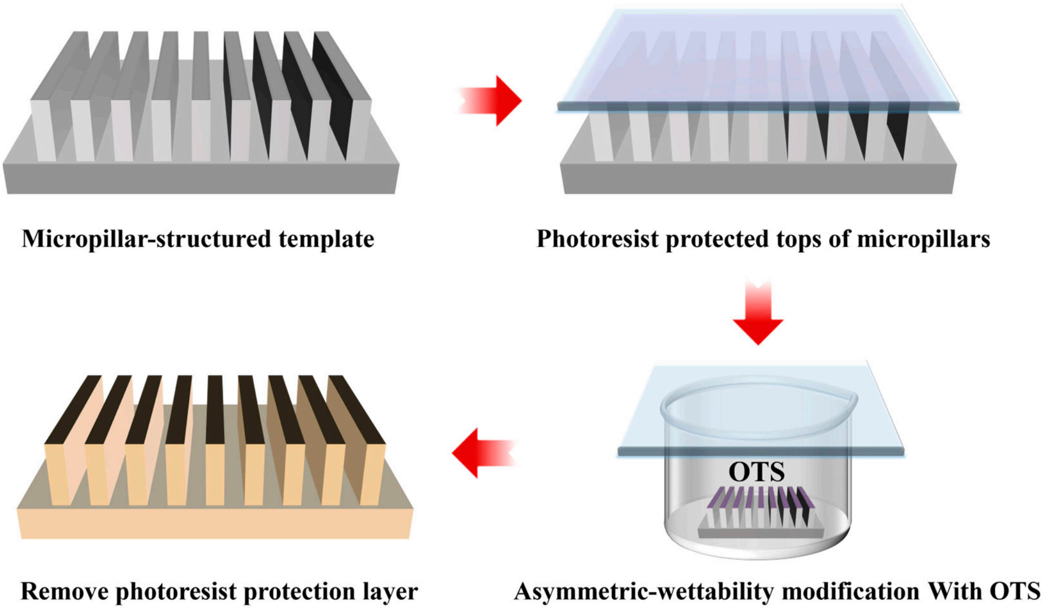

Figure S3. Schematic diagram for the asymmetric wettability modification of silicon templates with OTS.

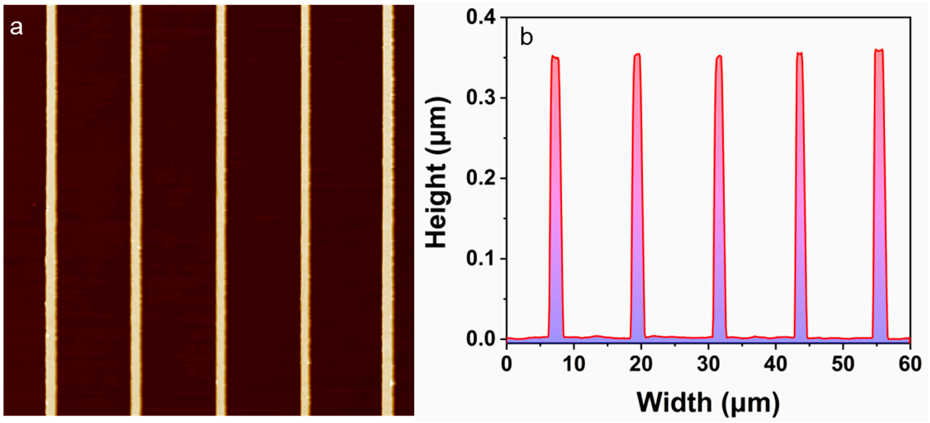

Figure S4. (a) AFM image of the HDA-BiI_5 single-crystalline nanowire arrays and (b) corresponding height curves.

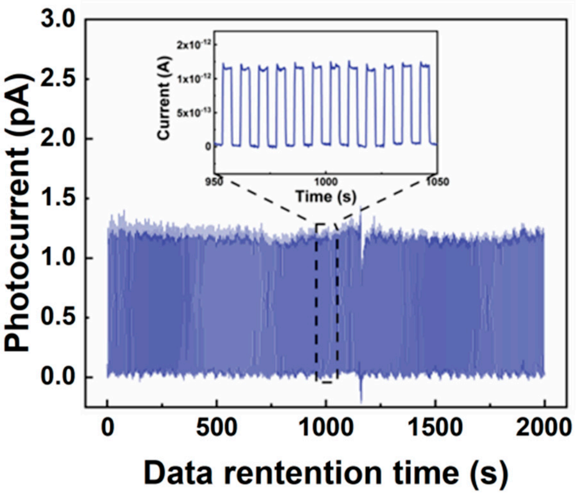

Figure S5. Endurance of capacitive optoelectronic memory devices based on the HDA-BiI_5 single crystal nanowires.

Disclaimer/Publisher's Note: The statements, opinions and data contained in all publications are solely those of the individual author(s) and contributor(s) and not of MDPI and/or the editor(s). MDPI and/or the editor(s) disclaim responsibility for any injury to people or property resulting from any ideas, methods, instructions or products referred to in the content.
